# Supplementary material for: Preparation, Characterization of New Antimicrobial Antitumor Hybrid Semi-Organic Single Crystals of Proline Amino Acid Doped by Silver Nanoparticles
Source: Biomedicines. 2023 Jan 26;11(2):360. doi: 10.3390/biomedicines11020360 (PMC9952970; doi:10.3390/biomedicines11020360)
Supplement: Supplementary file 1 [file biomedicines-11-00360-s001.zip › biomedicines-2039266-supplementary.pdf]

Table S1: Assigned vibra  tional FTIR bands spectra of crystals [22].

| Band | Assignment | Band | Assignment |
|---|---|---|---|
| 3430 (br) | υ(OH) | 1357(Sh) | $υ_{sCOO-}$ |
| 3066 (S) | υ($NH_2^+$) | 1318(w) | $w_{CH2}$ |
| 2983(Sh), 2789 (m) | $υ_{as}$($CH_2$) pyrrolidine ring | 1290(vs), 1253 (m) | $υ_{C-O}$ |
| 2504(Sh), 2381 (Sh) | | 1312(w) | $υ_{C-N}$ |
| 2642(m) | $υ_{CH}$ | 1164(w) | $t_{CH2}$ |
| 1623 (S) | $δ_{NH2+}$ | 1023(m) | $ρ_{CH2}$ |
| 1559(S) | $υ_{asCOO-}$ | 834(m) | $γ_{CH2}$ |
| 1487(m) | $υ_{C-N}$ | 871(w) | $γ_{NH2+}$ |
| 1454(m), 1402(Sh), 1367(Sh) | $δ_{CH2}$ | 589(w), 538(w) | $δ_{CH}$ bending in plane |
| 1458(m) | $δ_{NH}$ | 496(m) | $ρ_{C=O}$ |

υ, stretching; δ, in-plane bending; γ, out-of-plane bending; *w*, wagging; *t*, torsional; *ρ*, rocking; v, very strong; s, strong; w, weak; m, medium; br, broad; sh, shoulder; as, asymmetric; s, symmetric.

### SI.1. Shape index symmetry method

For peak "S" , ratio of slopes of curve tangents at inflection points (a/b) is a function of reaction order, n ($1^{\circ}$, $2^{\circ}$ order, …. etc.) and is determined from of DTA curve [50].

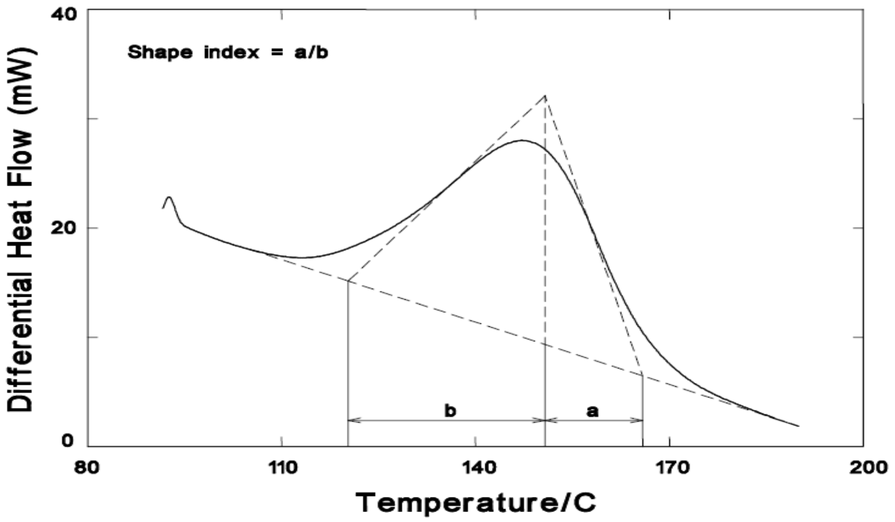


$$S = 0.63n^2 \quad (2)$$

$$n = 1.26\sqrt{\frac{a}{b}} \quad (3)$$

Decomposed substance fraction, $\alpha_m$, at maximum of decomposition reaction ($T=T_m$) is determined using relation:

$$1 - \alpha_m = n^{1/(1-n)} \quad (4)$$

Kinetic parameters are calculated using Horowitz-Metzger equation:

$$Z = \frac{E_a}{RT_m}\phi\, exp\left(\frac{E_a}{RT_m{}^2}\right) = \frac{kT_m}{h}\; exp\left(\frac{\Delta S^{\#}}{R}\right) \quad (5)$$

Where, $\Delta S^{\#}$ entropy of activation, Z collision number, R universal gas constant 8.314 $J.mol^{-1}.k^{-1}$, $E_a$ activation energy, $\phi$ heating rate ($K.s^{-1}$), k Boltzmann constant and h Planck's constant.

Heat of transformation enthalpy change, $\Delta H^{\#}$ is calculated from DTA curves. for phase transformation at peak temperature $T_m$ is given by equations:

$$\Delta H^{\#} = \frac{\Delta S^{\#}}{T_m} \quad (6)$$

Arrhenius relation, Fig.S1.

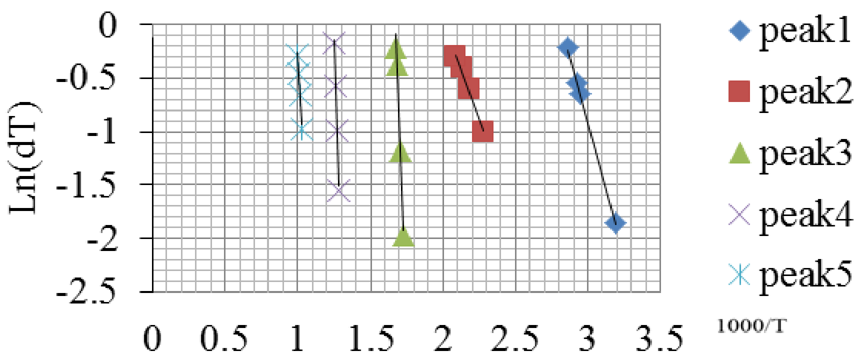


**Fig.S1:** ln ΔT against $10^3$/T relation for $[Co(L_1)_2(H_2O)_2].6H_2O$

Table S2: Bond length, Å and bond angle° of PCC

| Bond | Bond Length, Å | Bond | Bond Length, Å | Bond | Bond Length, Å |
|---|---|---|---|---|---|
| Co(1)– O(3) | 1.92924 | Co(1) – O(2) | 1.91977 | Co(2) – O(8) | 1.91895 |
| Co(1)–N(6) | 1.97529 | Co(1) –N(1) | 1.96350 | Co(2) –O(9) | 2.28705 |
| Co(1)–O(7) | 2.31463 | Co(1)–O(3) | 2.30251 | Co(2)–N(4) | 1.96080 |
| Co(1)–O(12) | 2.29467 | Co(1)–O(4) | 2.28340 | Co(2)–N(3) | 1.94554 |
| Co(1)–N(14) | 1.95821 | Co(1)–N(2) | 1.94534 | Co(2)–O(10) | 2.30472 |
| Co(1) –O(16) | 1.91632 | Co(1) –O(5) | 1.90705 | Co(2) –O(11) | |

Dihedral angles°

| Bond | Bond angle° | Bond | Bond angle° | Bond | Bond angle° |
|---|---|---|---|---|---|
| O3-Co1-N6 | 94.36 | N6- Co1- O16 | 84.68 | O12- Co1- N6 | 86.02 |
| O3-Co1- O7 | 83.94 | O7-Co1- O12 | 179.09 | N6- Co1- N14 | 172.34 |
| O3-Co1- O12 | 96.97 | O7- Co1- N14 | 78.29 | N14-Co1-O16 | 95.74 |
| O3- Co1- N14 | 84.83 | O12- Co1- N14 | 101.63 | O7- Co1- N16 | 94.06 |
| O3-Co1- O16 | 177.00 | O7-Co1- O12 | 93.29 | O12-Co1- O16 | 85.81 |
